# Newborn screening- the roadmap for India

**DOI:** 10.1186/1755-8166-7-S1-I41

**Published:** 2014-01-21

**Authors:** Seema Kapoor

**Affiliations:** 1 Department of Pediatrics, Maulana Azad Medical College, New Delhi, India

## 

India witnessed a major transition in 2013. It marked the completion of 50 years of the activities of the Indian Academy of Pediatrics, the completion of a taskforce study conducted by ICMR, organization of the Asia Pacific meeting on newborn screening and inclusion of congenital hypothyroidism as an important activity of a landmark program by the Government, the Rashtriya Bal Suraksha Kayakram. It has also created a platform for inclusion of many Delhi Hospitals in newborn screening.

These activities have created 3 major thrust points in the country which include awareness about the need for screening, the feasibility of its execution in the metropolitan cities of our country and the commitment to make the special diets available. Both public and private players have developed deep commitments which are likely to change the face of newborn screening in the country. Screening for 5 nearly completely reversible and treatable disorders is possible. These include Congenital Hypothyroidism, Congenital Adrenal Hyperplasia, Glucose-6-phosphate dehydrogenase deficiency, Biotinidase deficiency and Galactosemia. These have been included in different pilot programs across India.

Challenges which invite discussion are the feasibility of coverage in less well developed areas, hilly terrains and the home deliveries. MCTS (Mother child tracking system) an initiative launched by the Government of India once in full implementation may play a pivotal role in this program. Availability of good counselors and a well integrated follow up system needs to be developed so that all screen positive babies can be followed up. Generation of epidemiologic data for the currently untreatable conditions being the non availability of diets needs to be simultaneously addressed so that we can gear up for the expanded phase later.

Generating ethnic cutoffs, ensuring quality compliance, improving availability of confirmatory tests needs to be addressed. The volumes are formidable but also suggest that with high rates of consanguinity and inbreeding one is likely to encounter a significant proportion of these in the country. The most positive aspect is the commitment to this noble cause which will help us cross and reach the horizon.

